# Burden of waterpipe smoking and chewing tobacco use among women of reproductive age group using data from the 2012–13 Pakistan Demographic and Health Survey

**DOI:** 10.1186/s12889-015-2433-7

**Published:** 2015-11-12

**Authors:** Muhammad Tahir Khan, Shahkamal Hashmi, Sidra Zaheer, Syeda Kanwal Aslam, Naveed Ali Khan, Hina Aziz, Nabil Rashid, Kashif Shafique

**Affiliations:** School of Public Health, Dow University of Health Sciences, OJHA Campus, SUPARCO road, Gulzar e Hijri, Karachi, Pakistan; Department of Surgery, Dow University of Health Sciences, Karachi, Pakistan; Institute of Health and Wellbeing, Public Health, University of Glasgow, 1-Lilybank Gardens, Glasgow, G12 8RZ UK

**Keywords:** Waterpipe, Chewing tobacco, Tobacco, Tobacco use by women, Reproductive age group

## Abstract

**Background:**

Despite the general decline in cigarette smoking, use of alternative forms of tobacco has increased particularly in developing countries. Waterpipe (WP) and Chewing Tobacco (CT) are two such alternative forms, finding their way into many populations. However, the burden of these alternative forms of tobacco and their socio demographic determinants are still unclear. We assessed the prevalence of WP and CT use among women of reproductive age group in Pakistan.

**Methods:**

Data from the most recent Pakistan Demographic and Health Survey 2012–13 (*n* = 13,558) was used for this analysis. Information obtained from ever married women, aged between 15 and 49 years were analyzed using two separate data subgroups; exclusive WP smokers (total *n* = 12,995) and exclusive CT users (total *n* = 12,771). Univariate and Multivariate logistic regression analyses were conducted and results were reported as crude and adjusted Odds Ratio with 95 % confidence intervals.

**Results:**

Prevalence of WP smoking and CT were 4 % and 2 %, respectively. After multivariate adjustments, ever married women who were: older than 35 years (OR; 4.68 95 % CI, 2.62–8.37), were poorest (OR = 4.03, 95 % CI 2.08–7.81), and had no education (OR = 9.19, 95 % CI 5.10–16.54), were more likely to be WP smokers. Similarly, ever married women who were: older than 35 years (OR = 3.19, 95 % CI 1.69–6.00), had no education (OR = 4.94, 95 % CI 2.62–9.33), were poor (OR = 1.64, 95 % CI 1.07–2.48) and had visited health facility in last 12 months (OR = 1.81, 95 % CI 1.22–2.70) were more likely to be CT users as well.

**Conclusion:**

Older women with lower socio-economic profile were more likely to use WP and CT. Focused policies aiming towards reducing the burden of alternate forms of tobacco use among women is urgently needed to control the tobacco epidemic in the country.

## Background

Despite the fact that the general trend in cigarette smoking is declining in many parts of the world, use of alternative forms of tobacco is either stable or on the rise globally [1–3]. Waterpipe (WP) smoking and chewing tobacco (CT) are two of such risk behaviors finding their ways into many populations [3–6]. WP is a device that heats the tobacco indirectly and the smoke passes over a container of water and delivers to mouthpiece through a pipe [4]. This device is available with different names like hukka, sheesha, narghile, arguileh and hubble-bubble but essentially the basic configuration and mechanism of tobacco utilization is similar in all these devices [7–9]. WP use is traditional to Middle East and Indian subcontinent [5, 10]. Unlike cigarette smoking, WP smoking is gaining popularity among females with reported prevalence as high as 41 % [5, 7, 11]. Similarly, another form of tobacco consumption among females is CT, and has the prevalence as high as 12 % in low income countries like India and Pakistan [7, 12, 13]. Different forms of smokeless tobacco are available which are used either nasally or orally. These consist of guthka, zarda, toombak, dry snuff and moist snuff (with different compositions of tobacco, lime, mint and other ingredients) [14]. The recent increase in the consumption of these alternative forms, especially among females of reproductive age, might be attributed to the false belief that these alternate forms of tobacco are less hazardous than the cigarette smoking [15–18].

Contrary to the popular belief that these two forms are not toxic compared to cigarette, it has been reported that WP smoking is associated with lung cancer, bladder cancer, esophageal cancer, nasopharyngeal cancer, oral dysplasia low birth weight in pregnant women and several periodontal diseases [4, 10, 19]. The exposure to WP smoke and other metallic carcinogens through inhaling may have potential to increase the risk of cancer [20, 21]. Since WP smoking has become a common practice in cafes, restaurants and social gatherings, the risk of communicable diseases may also increase due to the repetitive sharing of WP mouth piece among individuals [4, 22]. Similarly, CT has also been implicated in causing respiratory and cardiovascular diseases and oral cancers apart from numerous periodontal conditions [23, 24].

The most worrisome aspect is the social acceptance of these alternate forms of tobacco which is increasing especially among women. Social acceptance of alternate forms of tobacco is increasing and special arrangements are made for the availability of these products in social gatherings. [24, 25]. Due to widespread social acceptability, it may be assumed that quitting the alternative forms of tobacco would be much more difficult than quitting cigarette smoking [25, 26].

Although few regional studies with smaller samples have reported the burden of alternative forms of tobacco use, there is limited evidence generated from population-based studies. Furthermore, most studies reported the burden of these alternative forms of tobacco use among heterogeneous age groups of population with limited representation of reproductive age women. Additionally, with the lack of nationally representative estimates of burden of alternative forms of tobacco, there is very limited understanding of the determinants of these risks behaviors. Therefore, the present study was conducted to determine the burden of alternative forms of tobacco use (WP and CT) among women of reproductive age group using a nationally representative sample, and identify socio-demographic determinants associated with use of these alternative forms of tobacco.

## Methods

### Data source

For this study we used the data from the Pakistan Demographic and Health Survey (PDHS) 2012–13 (*n* = 13,558). PDHS 2012–13 is the third survey conducted in Pakistan under the umbrella of Demographic and Health Survey (DHS) program [27]. DHS is a worldwide health survey program with the objective to collect data on fertility, women empowerment, domestic violence, mother and child nutritional status, contraception and family planning. National Institute of Population Studies (NIPS) successfully completed the PDHS with technical support from ICF International and Pakistan Bureau of statistics. The financial support was provided by the United States Agency for International Development (USAID) [26].

### Study population

Ever married women aged 15–49 years, resident of Pakistan with the exceptions of residents of Federally Administered Tribal Areas (FATA) and Azad Jammu and Kashmir due to adverse law and order situation and administrative independence respectively.

### Survey sampling frame

The sampling frame consisted of Pakistani urban and rural areas, and covered the four provinces and Gilgit Baltistan region. Multistage stratified sample design was used. At first stage, 500 primary sampling units (PSUs) were identified from urban and rural areas. At the second stage of sampling, 28 households were selected at each sampling point, through systematic random sampling. A sample size of 14, 000 households were estimated to provide precise data for this survey. The final selection of households comprises of 6, 944 households in the urban and 7, 056 households in the rural areas. The survey was conducted in 498 areas, however 24 areas (mainly in Baluchistan province) were excluded due to adverse law and order situation.

### Data collection tool

Woman Questionnaire was used to collect the information from ever married women aged between 15 and 49 years. The content of the household women’s questionnaire was based on the model questionnaire that was designed by the MEASURE DHS program [28]. The questionnaire was modified after the consultation with research institutions, government and non-government organization to address the issues in the local context of Pakistan. The final questionnaires were further translated into regional languages to obtain the most valid information in a systematic way [25, 26]. The response rate was 93.1 %

Study variables: The dependent variable, waterpipe or chewing tobacco use was measured using the following questions

1) Do you presently smoke or use any (other) type of tobacco? and in case of assertion the next question was 2) What (other) type of tobacco do you currently smoke or use?

a) Pipe b) chewing tobacco/nuswar c) snuff d) hukka (waterpipe) ,e) or specify any other form used.

Since the participants had the choice of selecting more than one type of tobacco products in the above mentioned question, therefore only those participants were considered who reported exclusive use of WP [option d) hukka (waterpipe)] or chewing tobacco [option b) chewing tobacco/nuswar] to avoid duplication/overlap of participants. For current smokers, last 24 h use was asked.

Only those were considered users of WP or CT who exclusively reported using any of these products. The participants using two or more types of tobacco were excluded from the study to prevent any bias caused by factors associated with more than one form of tobacco use.

Independent variables were selected considering previously reported evidences [5, 11, 29, 30]. These included: respondents’ age, area of residence (rural/urban), education level, wealth index, access to media, health facility visit in last 12 months, and respondents’ employment status.

### Informed consent and ethical consideration

The survey was conducted under the umbrella of global “MEASURE DHS” program, and is the third survey from Pakistan. All ethical guidelines of the “MEASURE DHS” program were followed by the National Institute of Population Studies. However, any formal detail regarding the ethical approval by any committee is not available in the PDHS report [27]. The authors obtained data for secondary analysis following prescribed procedure on MEASURE DHS website. (URL: http://www.dhsprogram.com/data/available-datasets.cfm) .

### Data analysis

We constructed two separate data subgroups for analyses; exclusive WP smokers and exclusive CT users. The first subgroup consisted of 12,995 ever married women. In order to have specific information related to exclusive WP smoking, information from 518 participants was excluded due to the use of other forms of tobacco as well. The second subgroup, included 12,771 ever married women who were exclusive CT users (excluding 742 participants who used other forms of tobacco as well). Ninety seven participants were also excluded due to missing information on the following variables: reading newspaper/magazine (*n* = 43), listening to radio (*n* = 6), watching television (*n* = 6), visit to the health facility in last 12 months (*n* = 4) and women employment status (*n* = 38).

The PDHS 2012–13 followed the multistage stratified sampling design to collect survey data, and thus primary sampling units (PSU), stratum numbers, and strata final weights were used to perform Complex survey data analysis. Univariate and multivariate logistic regressions were used to examine the associations between the outcome variables and covariates. We performed multivariate logistic regression with all the covariates that were found to be significant in univariate logistic regression, except the place of residence with chewing tobacco users. In multivariate logistic regression, we adjusted for age, area of residence, highest education level, wealth index, access to media, health facility visits and women employment status. Results were reported as odds ratios with 95 % confidence intervals. We used SAS version 9.1.3 for data analysis.

## Results

Overall prevalence of WP smoking was 4 % and the prevalence of CT use was 2 %. Among WP smokers, 53.4 % were aged > 35 years, 62.3 % resided in rural settings, 85.6 % had no education, 61.7 % belonged to lowest wealth quintile, 62.5 % had access to media, 62.6 % had not visited health facility in last 12 months, and 70.1 % were not currently employed. Among CT users, 60.7 % were older than 35 years, 62.8 % were rural dwellers, 81.6 % had no education, 49.5 % belonged to lowest wealth quintile, 63.5 % had access to media, 18.4 % had not visited health facility in last 12 months, and 70.4 % were not currently employed (Tables 1 and 2).

**Table 1:** Coding plan for the selected study variables

Variable	Question In Codebook	Coding for analysis
Outcome Variables		
Exclusive waterpipe smokers	1. Do you presently smoke or use any type of tobacco? Options included: 1 = yes, 2 = no	Women who answered both ‘Yes’ to Qs. 1 and ‘ hukka (waterpipe)’ to Qs. 2 were labelled as exclusive waterpipe smokers, and women who answered both ‘Yes’ to Qs. 1 and ‘ chewing tobacco’ to Qs. 2 were labelled as exclusive chewing tobacco users, and those who answered ‘No’ to Qs. 1 were labelled as non-tobacco users. (Respondents had the option to report multiple responses for Qs. 2)
Exclusive chewing tobacco users	2. What type of tobacco do you currently smoke or use? Options included: 1 = cigarette, 2 = pipe, 3 = chewing tobacco, 4 = snuff, 5 = hukka (waterpipe), 6 = or specify any other form used.	
Independent Variables		
Age in years (categorical)	What was your age on last birthday?	0 = 15 to 24
		1 = 25 to 35
		2 = > 35
Place of residence	Options included: 1 = large city, 2 = small city, 3 = town, 4 = rural	Options 1, 2, and 3 were recoded as 0 = urban, 1 = rural
Highest education level	What is the highest class you completed?	0 = no education
		1 = primary
		2 = secondary/higher
Wealth index	Options included: 1 = poorest, 2 = poorer, 3 = middle, 4 = richer, 5 = richest	Response options 1, 2 were recoded as 0 = poorer/poorest
		Response option 3 as 1 = middle
		Response options 4, 5 were recoded as 2 = richer/richest
Access to media	Do you read a newspaper or magazine? Options included: 1 = daily, 2 = at least once a week, 3 = occasionally, 4 = not at all.	Access to media: women who answered ‘daily’, ‘at least once a week’ or ‘occasionally’ to at least one question were recoded as ‘1’. No access to media: women who answered ‘not at all’ to all three questions were recoded as ‘0’.
	Do you listen to the radio daily? Options included: 1 = daily, 2 = at least once a week, 3 = occasionally, 4 = not at all.	
	Do you watch television daily? Options included: 1 = daily, 2 = at least once a week, 3 = occasionally, 4 = not at all.	
Visit to health facility in last 12 months	In the last 12 months, have you visited a health facility for care for yourself (or your children)? Options included: 1 = yes, 2 = no	0 = no, 1 = yes
Women employment status	Aside from your own housework, have you done any work in the last seven days? (sell things, have a small business or work on the family farm or in the family business). Options included: 1 = yes, 2 = no	0 = no, 1 = yes

**Table 2:** Descriptive characteristics of women regarding waterpipe smoking and chewing tobacco use, PDHS 2012–2013

Characteristics		Waterpipe Smokers		Chewing Tobacco Users
		*n* = 522		*n* = 277
	Total	*n* (%)*	Total	*n* (%)*
Age in years (categorical)				
15–24	2554	54 (10.3)	2524	23 (8.3)
25–35	4984	189 (36.3)	4887	86 (31.0)
>35	5457	279 (53.4)	5360	168 (60.7)
Place of residence				
Urban	6155	197 (37.7)	6063	103 (37.2)
Rural	6840	325 (62.3)	6708	174 (62.8)
Highest education level				
Sec./Higher	4038	43 (8.3)	4017	21 (7.6)
Primary	1778	32 (6.1)	1776	30 (10.8)
No education	7179	447 (85.6)	6978	226 (81.6)
Wealth index				
Richer/Richest	5740	99 (19.0)	5732	90 (32.5)
Middle	2476	101 (19.3)	2427	50 (18.1)
Poorer/Poorest	4779	322 (61.7)	4612	137 (49.5)
Access to media				
No	3555	196 (37.5)	3468	101 (36.5)
Yes	9440	326 (62.5)	9303	176 (63.5)
Visit to health facility last 12 months				
No	3693	327 (62.6)	3423	51 (18.4)
Yes	9302	195 (37.4)	9348	226 (81.6)
Women’s employment status				
No	10,477	367 (70.1)	10,317	195 (70.4)
Yes	2518	155 (29.9)	2454	82 (29.6)

(%)* = column percentages

### Waterpipe smokers

Univariate logistic regression analyses showed that women who were older than 35 years were 4.83 times more likely to be WP smokers (OR = 4.83, 95 % CI 2.69–8.64). Furthermore women who, lived in rural areas (OR = 3.20, 95 % CI 1.89–5.40), had no education (OR = 22.87, 95 % CI 12.59–41.55), were poorest (OR = 7.06, 95 % CI 3.88–12.84) and currently employed (OR = 1.92, 95 % CI 1.27–2.88) were significantly more likely to be waterpipe smokers. Access to media (OR = 0.58, 95 % CI 0.36–0.91) and recent health facility visit (OR = 0.34, 95 % CI 0.21–0.54) were significant protective factors against WP smoking. After multivariate adjustments ever married women who, were older than 35 years (OR = 4.68, 95 % CI 2.62–8.37), were poorest (OR = 4.03, 95 % CI 2.08–7.81), had no education (OR = 9.19, 95 % CI 5.10–16.54) were significantly more likely to be WP smokers. However they were less likely to be WP smokers if they had visited health facility in last 12 months (OR = 0.36, 95 % CI 0.22–0.55) (Table 3).

**Table 3:** Factors associated with waterpipe smoking and chewing tobacco use, PDHS 2012–2013

Characteristics	Waterpipe Smokers				Chewing Tobacco Users			
	Crude OR	95 % CI	Adjusted OR	95 % CI	Crude OR	95 % CI	Adjusted OR	95 % CI
Age in years (categorical)								
15–24	1		1		1		1	
25–35	2.03****	1.33–3.10	2.32****	1.52–3.53	1.81*	0.96–3.44	1.77*	0.94–3.32
>35	4.83****	2.69–8.64	4.68****	2.62–8.37	3.61****	1.91–6.80	3.19****	1.69–6.00
Place of residence								
Urban	1		1		1		1	
Rural	3.2****	1.89–5.40	0.99	0.56–1.73	1.26	0.82–1.93	0.63**	0.39–0.99
Highest education level								
Sec./Higher	1		1		1		1	
Primary	4.77****	2.16–10.52	3.18***	1.38–7.31	2.43**	1.21–4.84	2.56**	1.23–5.30
No education	22.87****	12.59–41.55	9.19****	5.10–16.54	5.87****	3.47–9.91	4.94****	2.62–9.33
Wealth index								
Richer/Richest	1		1		1		1	
Middle	3.45****	1.98–6.01	2.13**	1.18–3.83	1.28	0.82–2.00	0.97	0.61–1.54
Poorer/Poorest	7.06****	3.88–12.84	4.03****	2.08–7.81	2.3****	1.52–3.47	1.64**	1.07–2.48
Access to media								
No	1		1		1		1	
Yes	0.58**	0.36–0.91	1.49*	0.93–2.37	0.57****	0.41–0.78	0.93	0.67–1.29
Visit to health facility last 12 months								
No	1		1		1		1	
Yes	0.34****	0.21–0.54	0.36****	0.22–0.55	1.68***	1.13–2.49	1.81***	1.22–2.70
Women’s employment status								
No	1		1		1		1	
Yes	1.92***	1.27–2.88	1.31	0.89–1.91	1.65***	1.16–2.33	1.20	0.84–1.70

*Crude OR* unadjusted odds ratios, *CI* confidence intervals

*Adjusted OR* odds ratios adjusted for all independent variables

*p-value ≤ 0.1

**p-value ≤ 0.05

***p-value ≤ 0.01

****p-value ≤ 0.001

### Chewing tobacco users

Univariate logistic regression analyses showed that ever married women who, were older than 35 years (OR = 3.61, 95 % CI 1.91–6.80), had no education (OR = 5.87, 95 % CI 3.47–9.91), were poor (OR = 2.30, 95 % CI 1.52–3.47), were currently employed (OR = 1.65, 95 % CI 1.16–2.33), had visited health facility in last 12 months (OR = 1.68, 95 % CI 1.13–2.49), were more likely to be CT users. Furthermore ever married women who had access to media (OR = 0.57, 95 % CI 0.41–0.78) were less likely to be CT users. After multivariate adjustments ever married women who, were older than 35 years (OR = 3.19, 95 % CI 1.69–6.00), had no education (OR = 4.94, 95 % CI 2.62–9.33), were poor (OR = 1.64, 95 % CI 1.07–2.48) and had visited health facility in last 12 months (OR = 1.81, 95 % CI 1.22–2.70) were significantly more likely to be CT users. However women who lived in rural area were less likely to use CT (OR = 0.63, 95 % CI 0.39–0.99). Access to media and women employment status was not significantly associated with use of CT (Table 3).

## Discussion

The study reports important findings related to the use of alternate forms of tobacco including CT and WP, among Pakistani women in reproductive age group. Regarding the prevalence of the alternate forms of tobacco use among Pakistani women, the results are comparable with findings of latest country report by the World Health Organization [31]. A recent study reported that the prevalence of WP use is lower than 1 % among majority of lower middle income countries [32], however, we found a higher prevalence among the vulnerable subpopulation of females belonging to reproductive age group in Pakistan. There are several aspects to be taken into consideration while examining prevalence of alternate forms of tobacco use among Pakistani females. Firstly, females are being targeted by the tobacco industries, and prevalence of tobacco use among females is on the rise globally [33]. This holds true for Pakistani context as well [34]. Furthermore, it has been noted that the contemporary women with egalitarian attitudes exhibit an altogether different pattern of health related behaviors [35]. Additionally, the alternate forms of tobacco use like WP are becoming more common among masses [36]. Although the tobacco use prevalence among females is lower than their male counterparts, the issues like targeted tobacco industry’s marketing approach, increasing use of alternate forms of tobacco like waterpipe, and growing egalitarian attitudes among females may lead to increased vulnerability of females to tobacco hazards [36, 37]. Thus, it calls for deeper understanding of the specific characteristics of the female population that may be associated with use of tobacco in general, and alternate forms of tobacco in particular.

Women with lower overall socio-economic profile had higher odds of using alternate forms of tobacco. The poor, illiterate, rural dwellers, and the ones who had not visited health facilities for any reason whatsoever within last 12 months, were more likely to have health risk behaviors of CT and WP smoking. The findings are consistent with results from other studies in the country and the region [38, 39]. As a signing member of the Framework Convention on Tobacco Control (FCTC), Pakistani legislation, and health policies are obligated to control and prevent the tobacco use among masses [31]. However, our study findings reflect that there has been slow progress in this regard. Existence of socioeconomic disparities in use of tobacco indicates that we have not yet been able to reach the very roots of the vulnerable subpopulations. Also, it might indicate that the major part of the tobacco control efforts are skewed towards cigarette cessation and alternate forms of tobacco that are gaining access to masses, have not been given due attention yet. The findings may be informative to improve the anti-tobacco efforts, tailored according to the contextual characteristics of the vulnerable subgroups among population.

We also found that older women had higher odds of using alternate forms of tobacco. WP and CT have traditionally been used in South Asian cultures for a very long time [36]. Although it holds true for the older Pakistani generation too, there may be an optimistic perspective as well. Although long standing cultural ties of tobacco use still hold ground, but the youth is starting to realize the health effects associated with tobacco use, and is thus refraining from using alternate forms of tobacco. There are however, several complexities associated with the issue. Although the traditional WP use may be on a decline among youth, but the newer re-emergent forms like modern Sheesha is gaining popularity and even invading the cultures and societies which once knew little of its existence [36]. So it might be the case that the younger generations are simply switching from the older conventional form of tobacco use (waterpipe) to the newer more popular version (Sheesha). The issue calls for further exploratory studies in order to understand the declining, and emerging forms of tobacco that continue to pose threat to human health.

We also explored the association of tobacco use with access to health services. Women who had visited a health facility for any reason whatsoever in the past one year, were understandably protected against WP use, indicating better health related behaviors. However, strangely enough, they were more likely to use CT if they had visited the health facility in past year. The results probably hint towards a more complex phenomenon. It may be assumed that they have developed some pathological condition related to CT use, but ignorantly continue to use CT as they may not be aware of the ill health effects associated with it. This may then also point towards the gaps in health services provision. The health service providers may have missed the opportunity to either educate them regarding ill health effects related to CT; or these females have not yet been able to successfully quit its use. The cross sectional nature of the study, and secondary data analysis, hampers our ability to comment further, as we are unable to establish any temporal association. Thus, we call for further exploration of the finding.

The findings of this study may provide useful information regarding the use of alternate forms of tobacco among Pakistani females. Nationally representative large sample, sound and robust methodological approach of the Demographic Health Surveys (DHS) [28], add to the strengths of the present study. To the best of our knowledge, this is the first study to report use and determinants of alternate forms of tobacco specifically among women of reproductive age group, using nationally representative data. Nevertheless, there are several limitations. Firstly, we used secondary data, and could not account for the missing information. However, given the large sample size of the national survey, the findings may hold true for the majority of population. Secondly, cross-sectional nature of the study limits the ability to establish causal association. Thirdly, data from PDHS 2012–13 are self-reported, although evidence suggests that results generated from such self-reported data are reliable [40]. Further, PDHS 2012–13 mainly focused on women of reproductive age group, and the possibility of under-reporting of WP and CT use cannot be ruled out. Even then, the results may be useful as it identified the correlates of alternate forms of tobacco use among target population.

## Conclusion

The study reports that prevalence of waterpipe smoking, and chewing tobacco among Pakistani women is around four and two percent respectively. It also notes that women with lower socio-economic profile, and ones who were older in age, were more likely to report the use of alternate forms of tobacco. The findings may be useful for improving anti-tobacco efforts at population level. It may be recommended that national level programs need to target the alternate forms of tobacco (waterpipe and chewing tobacco) used in the community for better control of tobacco use.

## Abbreviations

CT: chewing tobacco
DHS: Demographic and Health Survey
FCTC: Framework Convention on Tobacco Control
OR: odds ratio
PDHS: Pakistan Demographic and Health Survey
PSU: primary sampling units
WP: waterpipe
